# Is repeat fine needle aspiration required in thyroid nodules with initial benign cytology? Results from a large Irish series

**DOI:** 10.1186/s12902-022-01014-6

**Published:** 2022-04-15

**Authors:** Hafiz M. Zia-ul-Hussnain, Oratile Kgosidialwa, Carmel Kennedy, Mark Quinn, Emma Dolan, Paul Deignan, Mark Sherlock, Chris J. Thompson, Diarmuid Smith, James P. O’Neill, Arnold Hill, Mary Leader, Helen Barrett, Cliona Ryan, Frank Keeling, Martina M. Morrin, Amar Agha

**Affiliations:** 1 Academic Department of Endocrinology, Beaumont Hospital, Dublin, Ireland; 2 Department of Surgery, Beaumont Hospital, Dublin, Ireland; 3 Department of Histopathology, Beaumont Hospital, Dublin, Ireland; 4 Department of Radiology, Beaumont Hospital, Dublin, Ireland

**Keywords:** Benign, Thyroid nodule, Thy classification, Fine needle aspiration

## Abstract

**Background:**

Fine needle aspiration (FNA) cytology is the preferred method for assessing thyroid nodules for malignancy. Concern remains about the rate of false negative results. The primary aim of this study is to investigate the malignancy rate of thyroid nodules initially classified as benign (Thy 2).

**Methods:**

We retrospectively examined 658 nodules in 653 (429 female) patients between January 2013 to December 2017. All FNA biopsies (FNABs) were performed under ultrasound (US) guidance by a radiologist with expertise in thyroid pathology. Nodules were cytologically classified according to the UK Royal College of Pathologists guidelines. Decisions about further management were made at a regular thyroid multidisciplinary meeting. Follow up of the Thy 2 nodules was determined based on clinical and radiological criteria.

**Results:**

The mean age (± SD) was 53.2 (14.6) years. Five hundred out of 658 (76.0%) nodules were classified as Thy 2 (benign) after the first FNAB. Of these thyroid nodules initially classified as benign, 208 (41.6%) underwent repeat FNAB and 9 (1.8%) were surgically removed without repeat FNAB. The remainder were followed up clinically and/or radiologically. Seven (1.4%) of nodules initially classified as Thy 2 were later shown to be or to harbor malignancy after a follow-up of 74.5 (± 19.7) months. Papillary thyroid microcarcinomas were found co-incidentally in two thyroid glands of benign nodules, giving a true prevalence of 5/500 (1.0%).

**Conclusions:**

With a well targeted FNAB, the false negative rate of an initial benign thyroid FNA is very low thus routine second FNAB is not required in patients with a thyroid nodule initially deemed benign. Multidisciplinary input is imperative in informing decision making.

## Background

The prevalence of thyroid nodules is population dependent, increases with age and depends on mode of diagnosis [1].Prevalence of thyroid nodules is estimated to be approximately 4% by palpation [2] but as high as 67% by thyroid sonography [3]. The malignancy rate in non-functioning thyroid nodules is up to 5% [4, 5]. The risk of malignancy in thyroid nodules classified as highly suspicious on thyroid ultrasound (TUS) is estimated at 70–90% compared to less than 1% of nodules reported as possessing mainly benign features [6]. However, a significant percentage of nodules will be indeterminate on TUS and will require a fine needle aspiration biopsy (FNAB) for a more definitive diagnosis. While ultrasound guided FNAB with direct visualisation reduces the false-negative rate, it has been reported to be as high as 6% in some series [7, 8], in those nodules previously reported as benign at repeat cytology.

The British Thyroid Association (BTA) recommends using the Ultrasound (U) classification to give guidance on nodules that may need further fine needle aspiration (FNA) to rule out malignancy [9]. Other international societies and scientific bodies use a comparable system of risk stratification [6, 10]. The BTA also recommends using the Royal College of Pathologists (RCPath) Thy Classification system (Thy classification) to standardise reporting of cytology results. Both the BTA and American Thyroid Association (ATA) recommend repeat TUS with FNA on sonographically and/or clinically highly suspicious nodules after initial benign cytology [6, 9]. In addition, the ATA recommends that follow-up of nodules with an initial benign cytology should be risk stratified according to TUS pattern [6].

Debate remains active whether radiologically or clinically defined low risk thyroid nodules that meet the criteria for FNAB require repeat FNA when initial cytology is benign (Thy 2). We previously reviewed our cohort of patients with benign reported thyroid nodules using routine repeat FNAB and found a low risk of malignancy after first benign cytology [4].

The primary aim of this study is to extend our previous study to investigate the malignancy rate of nodules classified as benign on initial FNAB.

## Methods

This was a retrospective cohort study of consecutive patients who had undergone FNAB of a thyroid nodule between January 2013 and December 2017 in a tertiary referral institution. All patients who had undergone FNAB within this time period were identified from the pathology data base WINPATH. Patient details were acquired from their medical notes and the electronic patient laboratory system. TUS reports were accessed through the hospital radiology system. All FNAB results were reported according to the UK Royal College of Pathologists Thy Classification System [11]. Samples classified according to the Thy classification system are summarised as follows; Thy 1 are non-diagnostic, Thy 2 are non-neoplastic, Thy 2c are non-neoplastic cystic samples containing abundant colloid, Thy 3a are samples containing atypical features but not enough to place into any of the other categories, Thy 3f are suspicious of follicular neoplasms, Thy 4 are suspicious of malignancy and Thy 5 are diagnostic of malignancy. Patients with a known history of thyroid malignancy and cytology not reported by our inhouse cytopathologists (within the study period) were excluded. Approval for the study was acquired from the hospital research and audit committee (Reference CA473).

### Decision making

Clinical decisions on management of patients were made at a bi-monthly thyroid multidisciplinary team (MDT) meeting. This was attended by specialists from endocrinology, cytopathology, endocrine surgery, radiology, chemical pathology and radiation oncology. Nodules with a Thy 2 classification were discussed at the thyroid MDM if there were any concerning clinical or radiological features or if the cytologist raised any concerns about the sample adequacy or recommended MDM discussion.

### Thyroid FNAB

All ultrasound guided thyroid FNABs were performed by one of two radiologists trained in TUS guided FNA, both of whom had over 20 years’ experience in sampling thyroid nodules. For most patients, thyroid nodules were reported using the Society of Radiologists in Ultrasound guidelines [12] before the introduction of U classification in 2016 in our institution. In a small subset of patients, thyroid nodules were reported according to the BTA U classification [9]. FNAB was routinely performed on U3 nodules ≥ 1.5 cm and U4/U5 nodules over 1 cm in maximum diameter. When a U classification was not available, nodules over 1–1.5 cm had an FNAB if the report qualitatively suggested an indeterminate or suspicious nodule. Thyroid cytology was reported using the Thy classification [11] by a small team of cytopathologists with a special interest in thyroid aspirates.

Repeat FNAB following an initial Thy 2 cytology was performed in the following situations;

1. The nodule showing new worrisome features on repeat TUS
2. In case of a nodule initially radiologically classified as U4 or U5 or reported as showing suspicious features.
3. If the nodule showed change in size of 20% or more in two dimensions after a follow up TUS.
4. Clinical discretion. For example, prior exposure to ionising radiation and family history of thyroid cancer.

### Assays

Thyroid stimulating hormone (TSH) was measured using the Elecsys electrochemiluminescence immunoassay (normal range 0.27–4.20 mIU/L). The free T4 (fT4) was measured using the Elecsys electrochemiluminescence immunoassay (normal range 12‑22 pmol/L).

### Statistical analysis

Data analysis was performed using IBM SPPS software version 24. Categorical data was reported in proportions and percentages. Continuous data was reported in mean and ± standard deviation (SD) for symmetrical data and median and range for asymmetrical data.

## Results

Six hundred and fifty-three patients (658 nodules) had undergone FNAB between January 2013 to December 2017. Initial cytological classification following the first FNAB and thyroid nodule size are shown in Table 1. Five patients had two nodules biopsied at the same time. After the first FNAB, the most commonly reported cytological classification was Thy 2 (benign) (76.0%). There was a trend for higher risk nodules to be larger in size, although this difference was not statistically significant.

**Table 1:** Thyroid nodule cytological classification following the first FNAB and size; *p* = 0.38

Thy class	Number of nodules biopsied (%)	Size, Mean (± SD) (cm)
Thy 1	88 (13.4)	2.7 (1.2)
Thy 2	500 (76.0)	2.6 (1.1)
Thy 2c	30 (6.0%)	
Thy 3a	29 (4.4)	2.5 (1.3)
Thy 3f	19 (2.9)	2.2 (1.0)
Thy 4	5 (0.8)	3.1 (0.3)
Thy 5	17 (2.6)	3.2 (1.4)
Total	658 (100)	2.6 (1.2)

Baseline characteristics of patients with initial Thy 2 nodules are presented in Table 2. Baseline thyroid function tests (TFTs) were available for 394 patients (79.6%). The mean follow-up period was 74.5 (± 19.7) months. The last follow-up date was December 2020.

**Table 2:** Baseline characteristics of patients with initial Thy 2 nodules

	**All Patients with Initially Thy 2 Nodules ** ***N*** ** = 495**	**Patients with Thy 2 Nodules that Underwent Repeat FNAB ** ***N*** ** = 206**
Age, Mean (± SD) years	53.3 (14.4)	52.8 (13.9)
Female, N (%)	429 (86.3)	179 (86.1)
Ethnicity, N (%)		
White	478 (96.6)	205 (98.6)
Other	6 (1.2)	1 (0.5)
Unknown	11 (2.2)	2 (1.0)
Personal history of non-thyroidal cancer, N (%)	9 (1.8)	0 (0%)
Baseline fT4, Mean (± SD) pmol/l	11.1(3.2)	11.1 (4.4)
Baseline TSH, Median (± Min–Max) mIU/L	1.1(0.01- 12.8)	1.2 (0.01–12.8)
Baseline thyroid status, N (%)		
Euthyroid	370 (74.7)	152 (73.1)
Hyperthyroid	4 (0.8)	1 (0.5)
Subclinical hyperthyroid	13 (2.6)	2 (1.0)
Subclinical hypothyroid	4 (0.8)	2 (1.0)
Follow-up, Mean (± SD) time months	74.6(19.6)	85.4 (14.2)

*FNAB* Fine needle aspiration biopsy, *SD* Standard deviation, *fT4* Free thyroxine, *TSH* Thyroid stimulating hormone

### Management of Thy 2 (Benign)Thyroid Nodules

**Fig. 1:**
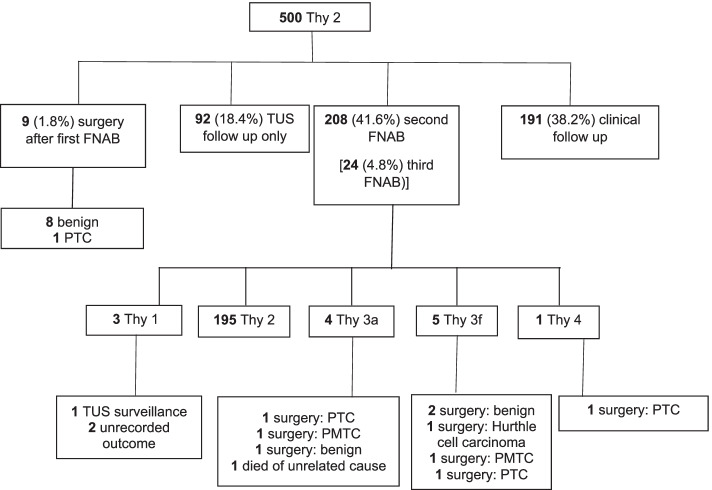
Management of patients with thyroid nodules initially classified as Thy 2. TUS Thyroid ultrasound, FNAB Fine needle aspiration biopsy, PTC Papillary thyroid cancer, PMTC Papillary thyroid microcarcinoma.

The management of nodules initially classified as Thy 2 are shown in Fig. 1. Of the 500 nodules initially classified as Thy 2, 208 (41.6%) and 24 (4.8%) underwent a second and third FNAB respectively (based on the aforementioned criteria). The rest of the nodules that were deemed low risk were followed up either by TUS (*n* = 92) or clinically (*n* = 191).

**Fig. 2:**
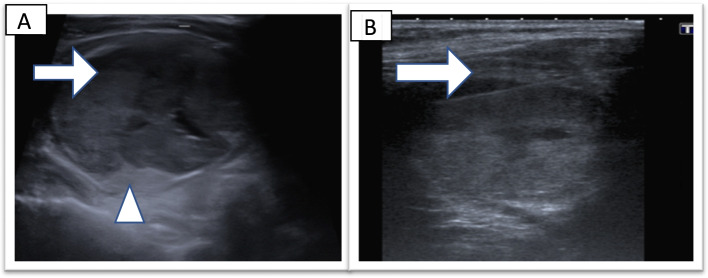
Thyroid ultrasound for patient 5; a 66 year old female with an incidental thyroid nodule identified while undergoing a carotid doppler ultrasound examination as part of a workup for transient ischaemic attack. Thyroid ultrasonography showing a 3.5 cm heterogeneous nodule with hypoechoic (arrow) and hyperechoic components (arrowhead) U3 (2A). Thyroid ultrasonography performed at the time of FNAB showing needle track advanced into the nodule (arrow) (2B)

**Fig. 3:**
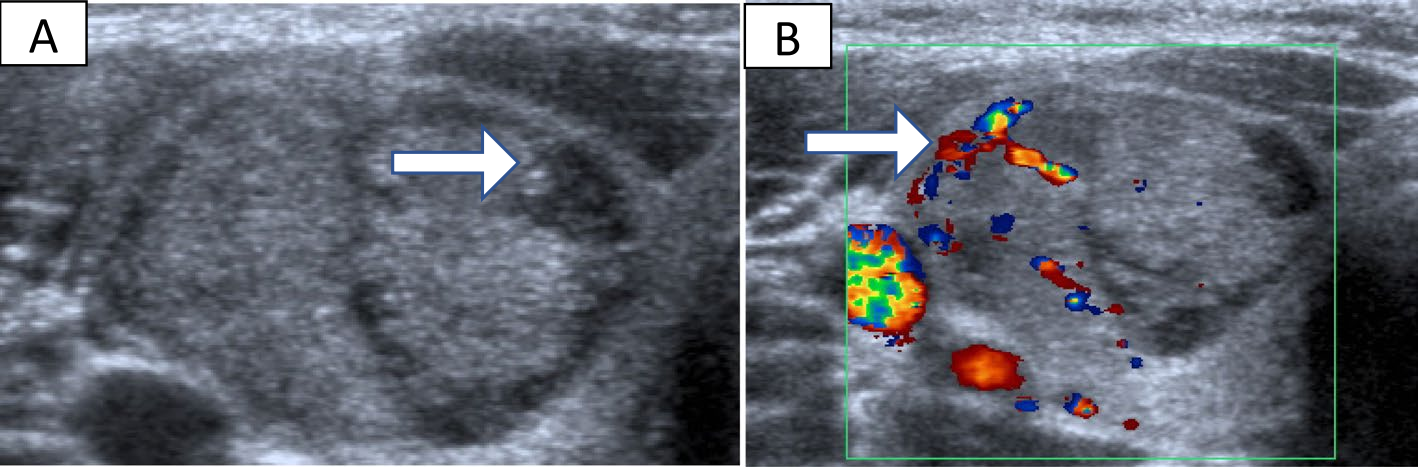
Thyroid ultrasound for patient 6; a 20 year old female who presented with a neck lump. Thyroid ultrasonography showing a 3 cm × 2.2 cm heterogenous thyroid nodule with focal areas of hypoechogenicity (arrow) (3A) and areas of vascularity (arrow) within the nodule (3B) (U3)

Seven (1.4%) patients initially classified as Thy 2 were found to have thyroid cancer following a thyroid lobectomy or thyroidectomy. Six of these nodules had higher risk Thy reclassification following a repeat FNA and the other one had surgery based on nodule appearance despite initial Thy 2 classification. Their details are shown in Table 3. TUS images of patient 5 and 6 are shown in Fig. 2 and 3 respectively. Two of the seven patients (patients 3 and 4) had microcarcinomas which were co-incidental findings in otherwise benign nodules. Excluding these two nodules, the true clinically significant miss rate was 5 out of 500 or 1%. Three nodules were re-biopsied following increase in size of the nodule.

**Table 3:** Patients classified as Thy 2 on initial cytology who had underlying thyroid malignancy

Patient	Gender	Age (years)	TUS findings	Repeat Thy classification	Reason for repeat FNAB/ surgery	Final histology and staging
1	Male	40	5 cm solid nodule	-	Clinical discretion	PTC
2	Female	46	2.5 cm cystic nodule	3a	Increase in nodule size of cold nodule from 2.5 to 4.5 cm in 10 months	Multifocal PTC; pT3NxMx
3	Female	50	1.5 cm solid nodule	3a	Increase in nodule size from 1.5 to 2 cm in 6 months	Incidental PMTC; pT1aNxMx;
4	Female	46	4 cm U4 nodule	3f	Suspicious TUS findings	Incidental 2 mm PMTC
5	Female	66	3.5 cm solid nodule	3f	Increase in size from 3.5 to 4 cm in 6 months on a background history of breast cancer	Hurthle cell carcinoma
6	Female	20	3 cm nodule	3f	Suspicious TUS findings	Multifocal PTC; pT2NxMx; 25 mm
7	Female	39	1.4 cm complex nodule	4	Clinical discretion	Multifocal cystic PTC; pT1NxMx

*CT* Computed tomography, *TUS* Thyroid ultrasound, *PTC* Papillary thyroid cancer, *PMTC* Papillary thyroid microcarcinoma, *TNM* staging Tumour node metastasis staging

All patients with the diagnosis of thyroid cancer were deemed to be in biochemical and structural remission following subsequent treatment.

## Discussion

In this study, we evaluated the outcome from FNAC in a large cohort of patients presenting with thyroid nodules over a period of 5 years. Among a large series of 500 nodules that were initially classified as benign (Thy 2), seven (1.4%) had malignancy diagnosed on follow up. Of these seven nodules, two were incidental microcarcinomas elsewhere in the thyroid and not clinically significant.. Our results are reassuringly similar to those reported in other series including our own prior work [4, 13].

In this study, 76.0% of nodules were deemed benign on initial cytology which is similar to the rate reported elsewhere [4, 14]. This estimate has remained stable at least over the last 30 years [15].

The sensitivity of FNAC as a screening tool is unclear, given the confounding factors which include sample adequacy, cytological expertise, inter observer disagreement, the potential for human error and practitioner bias. In a prior smaller study by our institution where we routinely performed two FNACs six months apart, the false negative rates in nodules initially classified as Thy 2 was 1% [4]. However, other studies showed a false negative rate which range from 1 to 7% [16, 17]. In a recent systematic review, the risk of malignancy in Thy 2 nodules has been reported to be around 5% [18]. One study showed a moderate interobserver agreement for Thy 2 nodules where cytology was reviewed by experienced members of the RCPath working group [19].

Repetition of FNAC and the use of ultrasound guidance may improve the sensitivity of FNAC as a screening tool for thyroid nodules. For example, in a sample of 306 patients with initially benign cytology, Orlandi et al. found malignancy in one and three patients on the second and third repeat biopsy respectively [13]. For this reason, they concluded that at least three FNACs of a thyroid nodule should be performed in order to fully exclude malignant thyroid pathology but this approach is clearly not practical at least for the majority of cases considering the very high volume of thyroid nodules routinely diagnosed clinically and radiologically. Others recommend a second biopsy only in patients with suspicious nodules as routine repeat FNAB on clinically stable disease were not found to be useful [16]. Currently, most societies do not recommend repeat FNAB in thyroid nodules with initial benign (Thy 2) cytology results and non-suspicious TUS findings unless there is a strong clinical suspicion of malignancy [6, 9].

A number of studies suggest that repeating FNAB on thyroid nodules that remain unchanged in size and ultrasound characteristics may not be cost effective, given the low rate of false-negative result following benign diagnosis on initial FNAC [16, 20]. Increase in size of a thyroid nodule, however, is not a reliable predictor of malignancy [21, 22]. In our current study, all nodules that were deemed malignant after an initially benign FNAC were ≥ 1 cm. In addition of these seven nodules, only three (including one PMTC) had shown increase in size. In this study, two nodules were found to harbor microcarcinomas that were co-incidental findings in otherwise benign nodules. PMTC are usually low risk, indolent tumors with little clinical significance although rarely they can show aggressive features [23, 24] and the management of these tumors remain controversial.

Reassuringly, nodules that are initially falsely classified as benign, do not seem to confer excess mortality. Nou et el who assessed a large cohort of 1365 patients with 2010 initial benign nodules, followed up for up for an average of 8.5 years showed that none of them died as a result of thyroid cancer despite identifying 18 false negative cases [25]. Others have reported similar findings [26]. Our data support these findings. This reassures the clinician that patients with initial benign thyroid cytology also have a low rate of disease specific mortality.

There are some limitations to our study. These include the retrospective nature of our study. In addition, 38.2% of the nodules were followed up clinically and 18.4% of the patients were followed up by TUS only. Therefore, malignancy cannot be completely ruled out in these nodules. However, this is real life experience and reflects the practice in most thyroid centres. The considerable follow up period of six years also means that it is unlikely that clinically significant cancers would have been missed.

## Conclusions

Our real-life experience of a large number of thyroid nodules shows a low rate of false negative thyroid cytology. When excluding incidental microcarcinomas and inadequate samples, the false negative result appears to be around 1%. Therefore, we recommend against routine repeat FNAC of low-risk thyroid nodules initially classified as Thy 2 by an experienced cytologist.

## Data Availability

The datasets used and/or analysed during the current study are available from the corresponding author on reasonable request.

## Abbreviations

ATA: American Thyroid Association
BTA: British Thyroid Association
CT: Computed tomography
FNAB: Fine needle aspiration biopsy
FNAC: Fine needle aspiration cytology
MDT: Multidisciplinary meeting
PMTC: Papillary thyroid microcarcinoma
PTC: Papillary thyroid cancer
ROM: Risk of malignancy
TSH: Thyroid stimulating hormone
TUS: Thyroid ultrasound
